# Mice carrying a complete deletion of the talin2 coding sequence are viable and fertile

**DOI:** 10.1016/j.bbrc.2012.08.061

**Published:** 2012-09-21

**Authors:** Emmanuel Debrand, Francesco J. Conti, Neil Bate, Lorraine Spence, Daniela Mazzeo, Catrin A. Pritchard, Susan J. Monkley, David R. Critchley

**Affiliations:** Department of Biochemistry, University of Leicester, Lancaster Road, Leicester LE1 9HN, UK

**Keywords:** MEFs, mouse embryo fibroblasts, FAs, focal adhesions, Integrin, Talin, Focal adhesions, Tln2 mouse knockout

## Abstract

Mice homozygous for several Tln2 gene targeted alleles are viable and fertile. Here we show that although the expression of talin2 protein is drastically reduced in muscle from these mice, other tissues continue to express talin2 albeit at reduced levels. We therefore generated a Tln2 allele lacking the entire coding sequence (Tln2^cd^). Tln2^cd/cd^ mice were viable and fertile, and the genotypes of Tln2^cd/+^ intercrosses were at the expected Mendelian ratio. Tln2^cd/cd^ mice showed no major difference in body mass or the weight of the major organs compared to wild-type, although they displayed a mildly dystrophic phenotype. Moreover, Tln2^cd/cd^ mouse embryo fibroblasts showed no obvious defects in cell adhesion, migration or proliferation. However, the number of Tln2^cd/cd^ pups surviving to adulthood was variable suggesting that such mice have an underlying defect.

## Highlights

► Mice lacking talin2 are viable and fertile with only a mildly dystrophic phenotype. ► Talin2 null fibroblasts show no major defects in proliferation, adhesion or migration. ► Maintaining a colony of talin2 null mice is difficult indicating an underlying defect.

## 1 Introduction

The cytoskeletal protein talin plays a pivotal role in regulating the activity of the integrin family of cell adhesion proteins and couples them to F-actin [1]. There are two talin genes that encode closely related (74% identity) proteins [2,3], but the relative roles of talin1 and talin2 are not yet understood. Tln1 is a relatively simple gene spanning approximately 30 kb, with 56 coding exons separated by relatively small introns. It is ubiquitously expressed and appears to give rise to a single major protein isoform. In contrast, Tln2, the ancestral gene [4], spans >400 kb, and although the genomic organization of the coding exons of the two genes is identical, the introns in Tln2 are much larger [2]. Moreover, Tln2 is a more complex gene with several promoters and splice variants. Talin2 is the predominant isoform in muscle and brain, but it is not present in most cells of haemopoietic origin.

Knockout or knockdown of talin1 in cultured cells leads to upregulation of talin2 which compensates for loss of talin1 [5,6]. However, in mice the functional relationship between the two proteins is less clear. Mice homozygous for deletion of the first four Tln1 coding exons die at about 8.5 dpc due to gastrulation defects [7], and hence, it had been assumed that talin2 was not expressed at this stage in development. However, talin2 has recently been shown to be present in embryonic epithelium at 7.5 dpc where it co-localises with talin1 and β1-integrin in cell/basement membrane junctions [8]. Nevertheless, it fails to compensate for loss of talin1 during the formation of the polarised elongated epithelial layer of the epiblast. Subsequent developmental processes appear to be less dependent on talin1, and conditional knockout of talin1 post 8.5dpc does not affect the development of most tissues/organs of the embryo other than the blood vessels, which fail to form properly due to defects in endothelial cell morphogenesis [9]. This is readily explained since endothelial cells express only talin1. The fact that deletion of talin1 does not affect development of other organs suggests that talin2 may be the functionally more important isoform in the majority of tissues.

To address the importance of talin2 during mouse development, we have used several gene trap alleles as well as a conditional allele in which the first coding exon of Tln2 is flanked by loxP sites. In all cases, mice homozygous for these various Tln2 alleles were viable and fertile arguing against a major role for talin2 in development [2,10]. However, the lack of isoform-specific antibodies made it impossible to establish whether such mice continue to express talin2 protein. Here we report the use of isoform-specific talin monoclonal antibodies [11] to show that talin2 is indeed expressed in some tissues in these mice suggesting that this may account for the lack of phenotype. We therefore generated a new Tln2 allele containing loxP sites at the 5′ and 3′ end of the first and last coding exons allowing complete deletion (cd) of the Tln2 coding sequence. Remarkably, although the Tln2 gene is widely expressed in wild-type mice, animals unable to express any talin2 exhibited no apparent developmental phenotype, and adult Tln2^cd/cd^ mice were viable and fertile, although they developed a mild form of muscular dystrophy similar to that previously reported using the exon1 deleted Tln2 allele [12].

## 2 Methods

### 2.1 Mouse strains

The following previously reported mouse strains were used in this study; Tln2^KO^ (Tln2tm1.1Crit) where Tln2 coding exon1 is deleted [12]; Tln2^sgt^ (Tln2Gt(S1-6D1)Sor) with a gene trap insertion upstream of the first coding exon [2,13]; Tln2^gt4^ (Tln2Gt(RRI434)Byg) with a gene trap insertion between coding exons 28 and 29 [2,13]. Mice (Tln2^cd/cd^) homozygous for complete deletion of the Tln2 coding sequence were generated in this study as described in Fig. S1 and Supplementary Methods.

### 2.2 Analysis of embryos, tissues and cells from Tln2^cd/cd^ mice

Talin1 and talin2 in embryos and tissues from genotyped animals were detected by Western blotting using the isoform-specific mouse monoclonal antibodies 68E7 and 97H6 respectively [11] as described in Supplementary Methods. Vinculin was detected using the mouse monoclonal antibody F9 (1:400), and FAK using an in house rabbit polyclonal antibody. Talin1 and talin2 in mouse embryo fibroblasts (MEFs) were visualised using the 68E7 and 97H6 antibodies as described by [11]. To determine rates of proliferation, cells were seeded on 6-cm dishes (10^5^ cells per dish) and counted every two days using a hemocytometer. Cell cycle distribution was determined on cells fixed in 70% ethanol, washed in PBS, treated with 0.5 mg/ml RNAseA (Sigma), stained with propidium iodide following the manufacturer’s protocol (e-Bioscience), and analysed using FAC SCanto™ II and FACS Diva software (BDBiosciences). Akt activation was assessed using confluent monolayers of serum-starved MEFs treated with 10% serum for 30 min. phospho-Akt (Ser-473) was detected by Western blotting with an antibody from Cell Signaling (Danvers, MA) diluted 1:1000. Bound primary antibody was detected with horseradish peroxidase-conjugated secondary antibodies (Sigma, 1:5000) and visualised using a SuperSignal West Femto ECL kit (Thermo Scientific). Immunohistochemistry on muscle was carried out as described previously [10,14].

## 3 Results

**Fig. 1:**
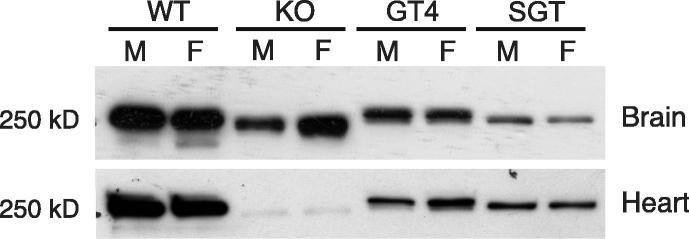
Talin2 protein is still expressed in current talin2 knockout mouse models. Western blotting for talin2 using the talin2-specific 68E7 monoclonal antibody [11] on brain and heart from wild-type (WT) male (M) and female (F) mice plus three Tln2 transgenic mouse lines; KO (exon1 deletion) [12]; GT4 (gene trap insertion in Tln2 coding sequence which generates a fusion protein containing the N-terminal half of talin2 fused to β-galactosidase [13], and SGT (gene trap insertion in 5′ UTR).

We have previously described a conditional Tln2 allele (Tln2^ko^) in which the first coding exon is flanked with loxP sites [12]. By crossing mice carrying this allele to the Cre deleter mouse, we generated mice that were homozygous for loss of the first Tln2 coding exon (Tln2^ko/ko^). The mice were viable and fertile, and Western blotting with a talin2 specific polyclonal antibody showed loss of talin2 protein from skeletal muscle resulting in a mildly dystrophic phenotype [12]. Here, we show that talin2 expression was also ablated in heart, but substantial amounts of talin2 were still detectable in brain (Fig. 1), spleen and kidney (data not shown), presumably due to alternative promoter usage or exon skipping. We also analysed talin2 protein expression in two Tln2 genetrap mouse lines that show no obvious phenotype. GT4 contains an insertion in Tln2 coding sequence that creates a fusion protein containing the N-terminal half of talin2 fused to β-galactosidase [13]. It is clearly expressed in brain and heart (Fig. 1) and kidney and spleen (data not shown), but at reduced levels compared to full-length talin2. SGT contains a gene trap insertion in the 5′UTR [2], and still expressed talin2 in heart (unlike the Tln2^ko/ko^ line) albeit at reduced levels (Fig. 1), and also in brain (Fig. 1), kidney and spleen (data not shown).

Since the lack of any developmental defects in these mice may be due to the continued expression of talin2 in some tissues, we decided to generate a truly null Tln2 allele (Tln2^cd^) by deleting the entire coding sequence. The targeting strategy is outlined in Fig. S1A. In brief, we took ES cells in which the first Tln2 coding exon was flanked by LoxP sites (Tln2^fl^ allele; referred to as the 5′ targeted allele in Fig. S1), and introduced an additional loxP site in the 3′UTR (exon 56) between the stop codon and the polyadenylation signal to generate a 5′ and 3′ targeted allele (Fig. S1A). Southern blotting confirmed that the 3′ end of the Tln2 gene was successfully targeted in ES clones 369, 374 and 397 (Fig. S1B). Each of these three ES cell lines were then transiently transfected with a Cre recombinase expression construct to delete any sequence flanked by loxP sites. The two possible outcomes are shown Fig. S1A. Where the 5′ and 3′ targeting events are in cis, then both the Tln2 coding sequence and the tyrosine kinase (TK) selection cassette will be completely deleted which will not be the case if the two targeting events are in trans. To identify the required ES cell lines, transfectants were cultured in the presence of ganciclovir and individual resistant sub-clones were screened by Southern blotting (Fig. S1B) and PCR (not shown). 6 clones (369.3, 369.5, 369.4, 374.1, 374.2 and 397.3) were identified in which the entire Tln2 coding region had been deleted.

**Fig. 2:**
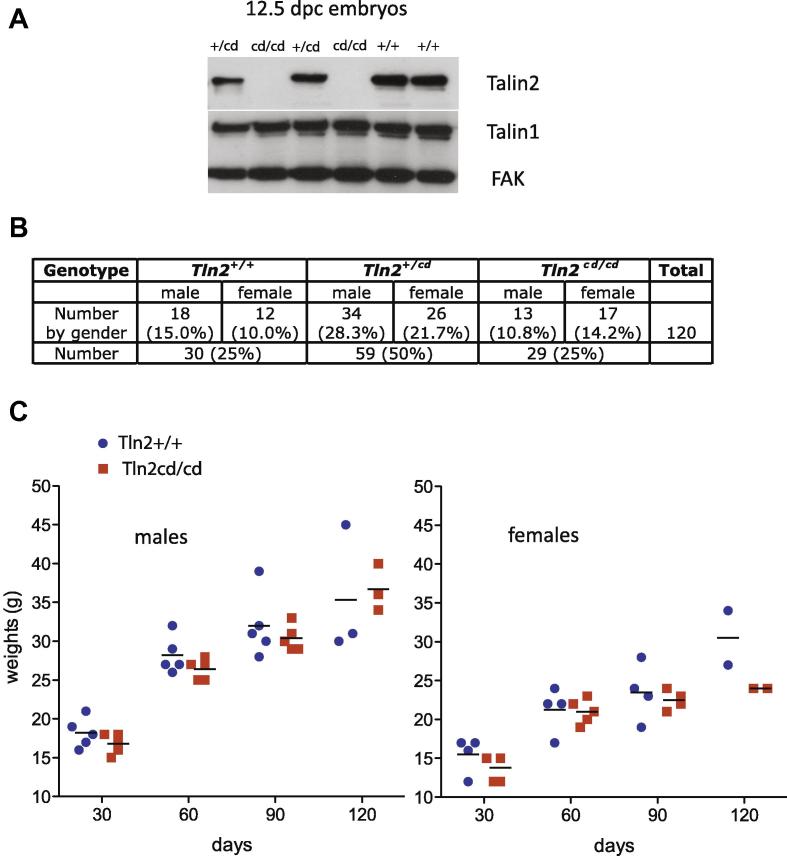
Talin2 null (Tln2^cd/cd^) mice possess no overt phenotype. (A) Tln2^cd/+^ mice were intercrossed and 12.5 dpc embryos of the genotypes shown screened for talin1 and talin2 by Western blotting. Tln2^cd/cd^ embryos contained no talin2 protein while levels of talin1 and focal adhesion kinase (FAK) were unchanged. (B) Tln2^cd/+^ intercrosses gave rise to the three possible genotypes at the expected frequency for both sexes indicating that the loss of talin2 does not result in embryonic or neonatal lethality. (C) Weights of male (left) and female (right) Tln2^cd/cd^ and Tln2^+/+^ mice plotted against days (blue circle, WT mice; red square, Tln2^cd/cd^ mice). The average weight for each group is displayed as a black bar. Both male and female Tln2^cd/cd^ mice tended to be smaller than their control littermates, but this was not statistically significant.

Mice were generated from some of these ES cell clones, and genotyped by PCR to confirm that they possessed the deleted Tln2^cd^ allele (not shown). Mice homozygous for complete deletion (Tln2^cd/cd^) appeared indistinguishable from their heterozygous and wild-type littermates. Analysis of lysates from 12.5dpc embryos confirmed that Tln2^cd/cd^ embryos had no detectable talin2 protein while Tln2^cd/+^ embryos showed a reduction in talin2 compared to wild-type (Fig. 2A).

**Fig. 3:**
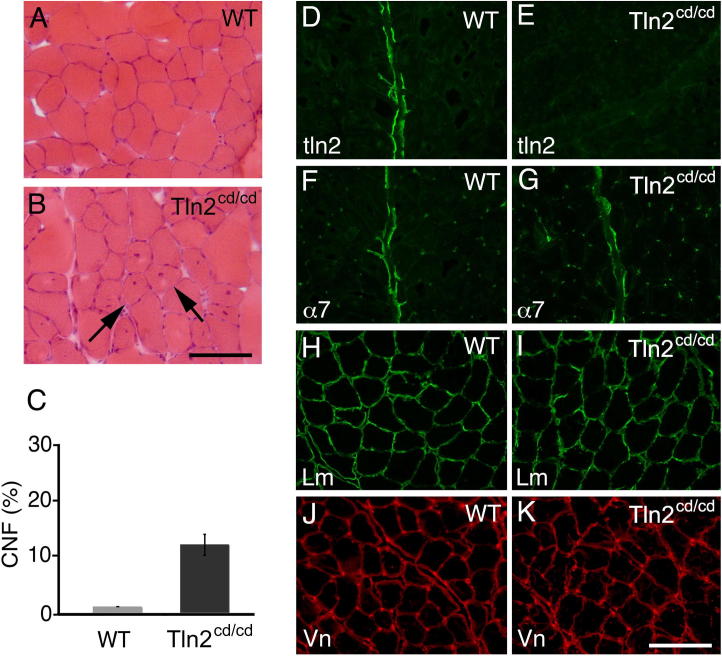
Tln2^cd/cd^ mice develop myopathy by 5 months of age. H&E stained gastrocnemius muscle from (A) wild-type (WT) and (B) Tln2^cd/cd^ mice. Arrows in (B) indicate centrally nucleated fibres (CNF). (C) Quantification of CNF in gastrocnemius muscle from Tln2^+/+^ (WT) and Tln2^cd/cd^ mice. Data represent the mean ± S.D.; ^∗∗^ indicates statistically significant differences (*p* = 0.004). (D–K) Immunostaining of WT and Tln2^cd/cd^ gastrocnemius muscle for talin2 (Tal2), α7-integrin (α7), laminin (Lm) and vinculin (Vn). Talin2 is expressed in the myotendinous junction of WT muscle but is absent from Tln2^cd/cd^ muscle. α7-integrin, which is also expressed at the myotendinous junction shows similar expression in both samples. Laminin (Lm) and vinculin (Vn) expression also remains unchanged in Tln2^cd/cd^ muscle. Scale bars = 50 μm.

Intercrossing Tln2^+/cd^ mice produced the expected Mendelian ratios of male and female wild-type, homozygous and heterozygous progeny (Fig. 2B). We also bred both male and female Tln2^cd/cd^ mice to wild type animals and these all produced litters of similar size and numbers compared to Tln2^+/cd^ mice. Thus, Tln2^cd/cd^ mice are viable and fertile. However, it was difficult to maintain a breeding colony of Tln2^cd/cd^ for unexplained reasons indicating that the mice have an underlying defect. We looked at the growth rates of Tln2^cd/cd^ compared to wild-type mice, and although the numbers were small, there was no clear difference in rate of weight gain after birth (Fig. 2C). Additionally there were no significant differences in the proportional weights of any of the major organs in Tln2^cd/cd^ mice compared to wild-type, either for males or females (Fig. S2), including the brain where talin2 is the predominant isoform [11]. The Tln2^cd/cd^ mice did exhibit a mild form of muscular dystrophy at 5 months as indicated by the marked increase in centrally located nuclei (Fig. 3), a phenotype very similar to that of Tln2^ko/ko^ mice which are only missing coding exon1 [12].

**Fig. 4:**
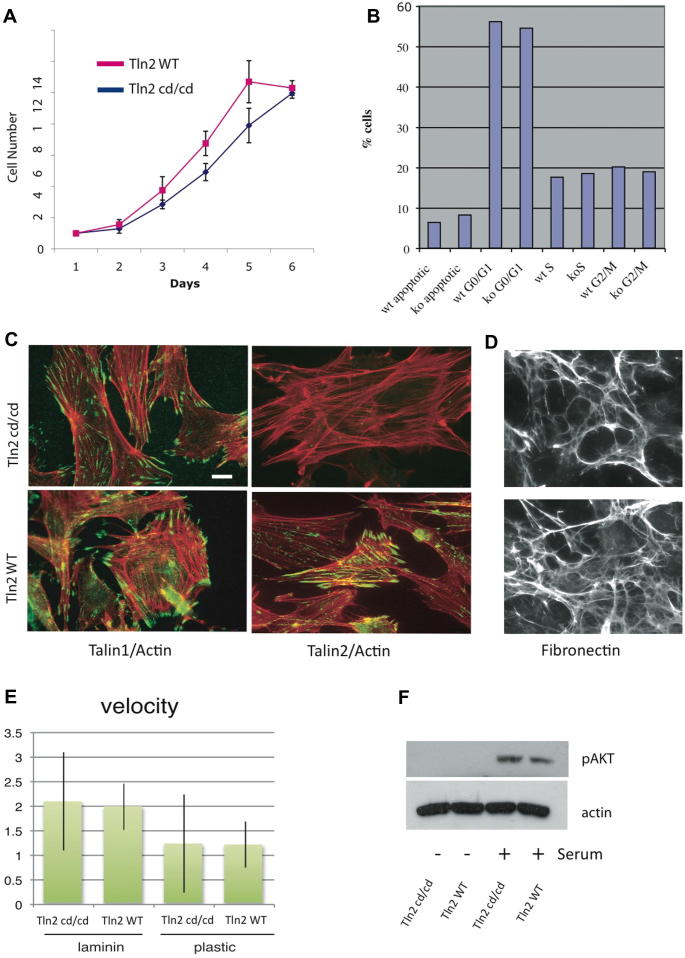
Tln2^cd/cd^ mouse embryo fibroblasts (MEFs) show no proliferation, adhesion or migration defects. (A) Tln2^cd/cd^ MEFs grew slightly slower than wild-type (WT) cells; (mean values ± S.D. (B) Cell cycle distribution of Tln2^cd/cd^ and WT MEFs was indistinguishable. (C) Tln2^cd/cd^ and Tln2 WT MEFs plated on plastic and stained for either talin1 or talin2 (green) and actin (red). Scale bar 10 μm. Talin2 null MEFs were able to spread and assemble talin1-containing focal adhesions and actin stress fibres, and also (D) assembled an extensive fibronectin matrix. (E) Tln2^cd/cd^ and WT MEFs showed similar rates of wound closure on either laminin or plastic. Line indicates range. (F) Akt activity in Tln2^cd/cd^ and WT MEFs was determined by Western blotting using a phospho473-AKT antibody. Serum-starved cells were stimulated with serum 30 min prior to analysis. (For interpretation of the references to colour in this figure legend, the reader is referred to the web version of this article.)

Talin2 null (Tln2^cd/cd^) mouse embryo fibroblasts (MEFs) isolated from 13.5dpc embryos (Fig. S3A) showed slightly reduced rates of cell proliferation compared to wild-type (Fig. 4A), although there were no obvious defects in cell cycle progression or any increase in apoptosis as indicated by FACS analysis (Fig. 4B) or Western blotting for PARP cleavage (data not shown). Tln2^cd/cd^ MEFs showed no obvious defects in cell spreading and assembled cell-extracellular matrix junctions (focal adhesions; FAs) and associated actin stress fibres despite the lack of talin2 protein (Fig. 4C). They also assembled an extensive fibronectin extracellular matrix (Fig. 4D), although we have previously shown that knockdown of talin2 in NIH 3T3 cells compromises matrix assembly [11]. Rates of wound closure on laminin-coated or uncoated tissue culture plastic were also unaffected by loss of talin2 (Fig. 4E and Fig. S3B).

Subsequent to generating the Tln2^cd/cd^ allele, it became apparent that we had also deleted the miR190 gene which is contained within the Tln2 gene. miR190 has recently been shown to suppress expression of Leucine Rich Repeat Protein Phosphatase PHLPP [15] which in turn negatively regulates AKT signaling. Loss of miR190 would therefore be predicted to result in upregulation of PHLPP and suppression of AKT signaling. However, AKT was activated to a similar extent in both Tln2^cd/cd^ and wild-type MEFs following addition of serum to serum-starved cells (Fig. 4F).

## 4 Discussion

Although mice homozygous for two Tln2 gene trap alleles [2,13] or a Tln2^ko^ allele [12] are viable and fertile, it is now apparent that these mice still express talin2 protein in several tissues. Thus, the lack of phenotype is difficult to interpret, and we therefore generated mice in which the whole of the Tln2 coding sequence was deleted. Surprisingly, the Tln2^cd/cd^ null mice were also viable and fertile, and inter-crossing Tln2^cd/+^ mice gave rise to the expected ratios of wild-type, hetero- and homozygous mice. Moreover, the weight of Tln2^cd/cd^ mice and their major organs was no different from wild-type animals. Similarly, preliminary analysis of blood and urine showed no obvious abnormalities.

Although Tln2 is reported to be the ancestral gene [4], the specific function of talin2 protein has not been established in any organism. Talin2 is more widely expressed than originally inferred from Northern blots [3], and it is the most abundant isoform in brain and muscle [11]. In vitro, talin2 is upregulated during myoblast fusion [16] suggesting an important role in muscle development, and talin2 co-localises with the muscle-specific β1D-integrin splice variant in myotendinous junctions [10,12]. Consistent with this observation, Tln2^cd/cd^ mice develop a mild form of muscular dystrophy characterised by disruption of the myotendinous junction, a phenotype very similar to that observed in mice homozygous for deletion of the first Tln2 coding exon [12]. Deletion of this exon results in a near complete ablation of talin2 expression in muscle, whereas in other tissues, talin2 continues to be expressed. Biochemical studies show that the N-terminal talin2 FERM domain binds β1D-integrin with higher affinity than talin1 [17], and the data reported here suggest that the tight binding of talin2 to β1D-integrin is important in stabilizing the myotendinous junction against the force exerted on it during muscle contraction. Talin1 on the other hand is predominantly localised in costameres, although there is some in myotendinous junctions. Consistent with this, muscle-specific knockout of talin1 gives rise to a less severe myopathy than that caused by talin2 knockout [10]. Interestingly, combined loss of talin1 and 2 from muscle leads to an embryonic lethal phenotype with defects in myoblast fusion [12]. Thus, in skeletal muscle, talin1 and talin2 appear to have distinct but overlapping functions.

Studies on cells in culture clearly establish that talin2 can compensate for loss of talin1, and talin2 can support cell spreading and FA assembly in talin1 knockout or knockdown cells [5,6]. However, as in muscle, the localization of the two proteins is subtly distinct [11] and Fig. 4C – talin1 is localised in FAs, while talin2 extends beyond FAs along actin stress fibres into fibrillar adhesions, structures involved in fibronectin matrix assembly. However, Tln2^cd/cd^ null cells showed no changes in the localization of talin1 nor any differences in cell spreading, FA formation, fibronectin matrix assembly or rates of wound closure. Given these findings, it is remarkable that germ line loss of talin1 results in embryonic lethality at gastrulation [7] despite the fact that talin1 and talin2 co-localise with β1-integrins in the basal aspect of the embryonic epithelium where cells contact the basement membrane [8]. In vitro studies show that although talin2 localisation in the embryonic epithelium is not affected by loss of talin1, both recruitment of paxillin to cell-matrix junctions and FAK signaling are both compromised, and the steady state level of β1-integrin was markedly reduced due to proteasomal-mediated degradation. Thus in the embryonic epithelium, talin1 rather than talin2 is required for epithelial cell polarisation and extension, the stabilization of β1-integrins and for integrin signaling.

Talin2 is not expressed in cells of hematopoetic origin [18] including platelets [19], and knockout of talin1 does not lead to upregulation of talin2 in these cells. In contrast, knockout of Tln1 in fibroblasts leads to the rapid upregulation of talin2 [6]. The major Tln2 promoter is embedded in a CpG island and it is presumably silenced in hematopoetic cells by methylation [2]. Little is known about the transcription factors that regulate talin2 expression [2] although Tln2 transcription is stimulated by the transcription factor YY1, an effect silenced by ERK-mediated YY1 phosphorylation [20] (Fig. S4). Talin2 expression is negatively regulated at the translational level by miR-132 [21] and also by the fragile X related protein FXR1 which binds to the 3′ end of the Tln2 mRNA and represses translation [22] (Fig. S4). Expression of the desmosomal protein desmoplakin is also regulated in the same way. Interestingly, knockout of FXR1 in heart leads to the upregulation of talin2 and desmoplakin proteins which compromises the integrity of costameres (where talin1 is localised) and desmosomes. Clearly, tight regulation of talin2 levels is essential to normal cardiac function, although the data reported here suggest that basal cardiac function is normal in Tln2^cd/cd^ mice.

A recently noted feature of the Tln2 gene is that it encodes the micro-RNA miR-190 which is contained within intron 51 towards the 3′-end of the gene [2]. Recent studies show that expression of both Tln2 and miR-190 are markedly increased by trivalent arsenic As^3+^ [15]. miR-190 down-regulates expression of the PH domain-containing leucine-rich repeat protein phosphatase PHLPP, which in turn negatively regulates the activity of Akt (Fig. S4). As a result, increased miR-190 expression enhances Akt signaling, and miR-190 over-expression stimulates cell proliferation and can lead to malignant transformation. Ablation of the miR-190 gene in the Tln2^cd/cd^ mice would be predicted to increase PHLPP phosphatase activity and thereby decrease Akt signaling. Down-regulation of Akt has the potential to affect numerous pathways, and could lead to p21-induced cell cycle arrest, increased Bax-mediated apoptosis and reduced mTOR-mediated translational initiation. miR-190 has also been implicated in the negative regulation of TGF-β signaling [23]. However, Tln2^cd/cd^ MEFs showed normal cell cycle progression although their doubling time was marginally slower than wild-type MEFs. Moreover, there was no evidence of increased apoptosis, and serum-stimulated AKT levels were comparable in the Tln2^cd/cd^ and wild-type MEFs. Overall, it is clear from these studies that neither talin2 nor miR-190 are essential for normal mouse development or the viability and fertility of adult mice, despite the fact that talin2 expression is tightly regulated at both transcriptional and translational levels. However, maintaining a breeding colony of Tln2^cd/cd^ mice is challenging for unexplained reasons, while Tln2^ko/ko^ mice in which the first codon exon has been deleted [12], and continue to express reduced levels of talin2 in non-muscle tissues, show no such problems. Complete loss of talin2, miR-190 or both may contribute to this phenotype.
