# Supplementary figures and images for: Mice carrying a complete deletion of the talin2 coding sequence are viable and fertile
Source: Biochem Biophys Res Commun. 2012 Sep 21;426(2-3):190–5. doi: 10.1016/j.bbrc.2012.08.061 (PMC3485561; doi:10.1016/j.bbrc.2012.08.061)

Fig S1

A

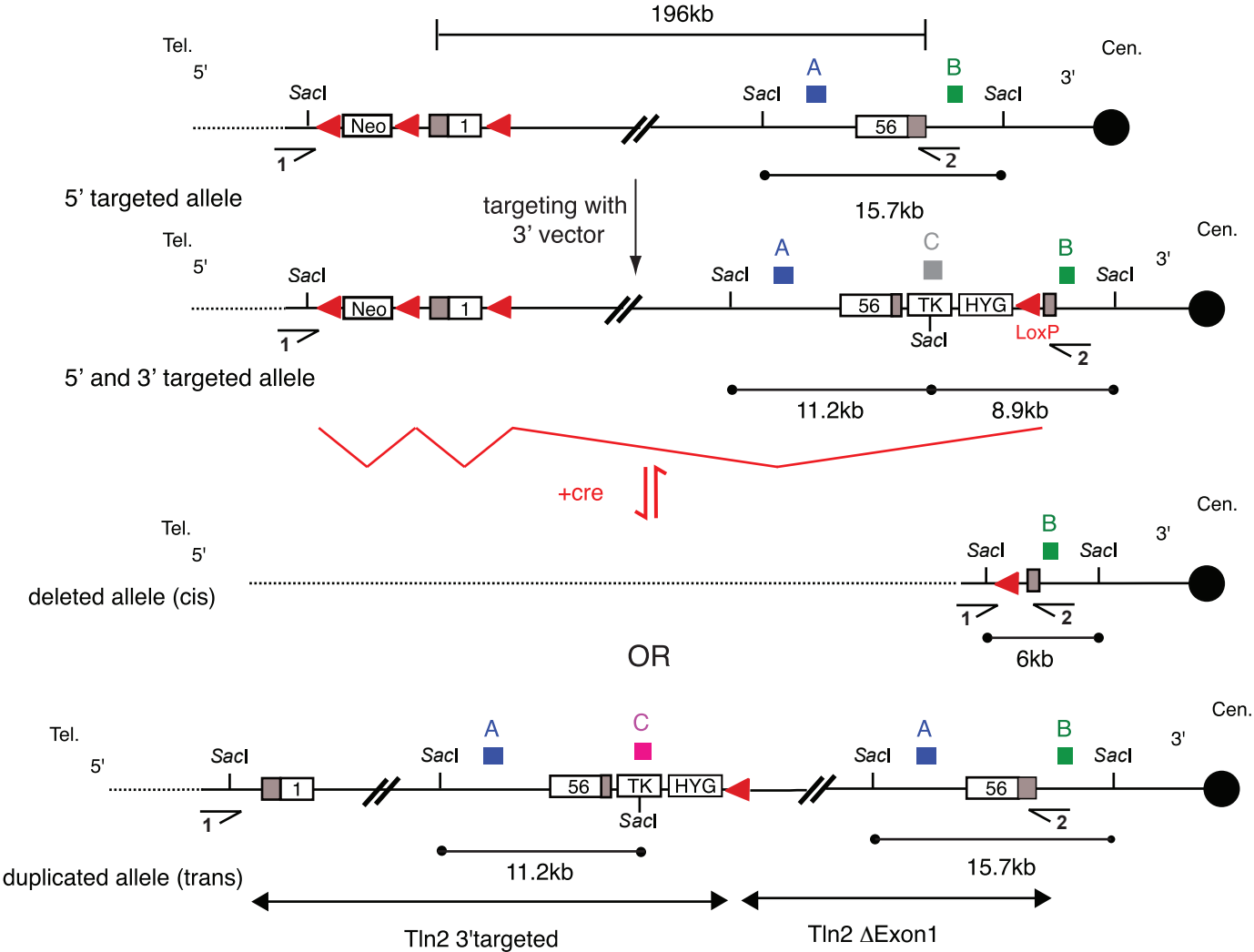

B

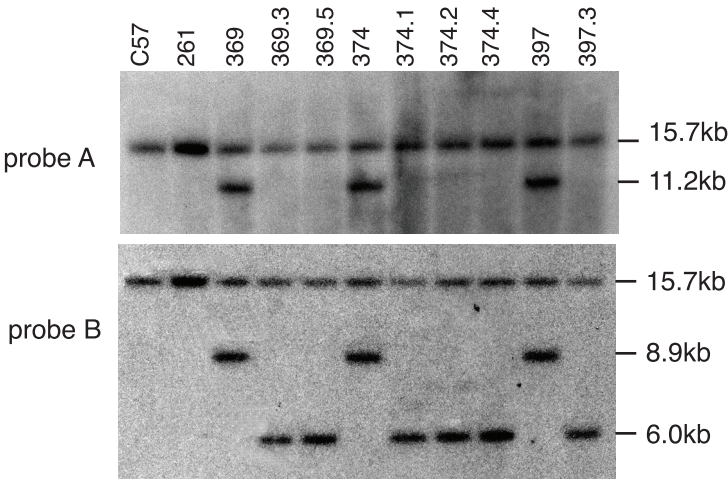

Supplement: Supplementary data 1 [file mmc1.pdf]

Fig S2

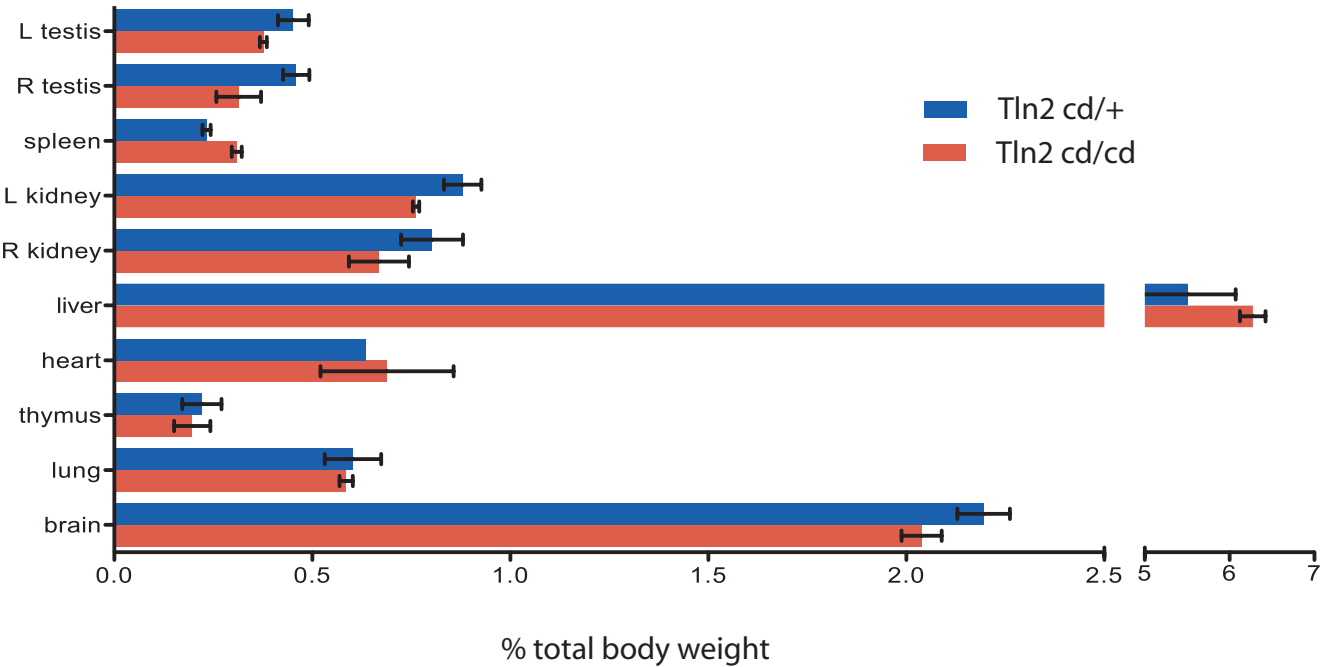

Supplement: Supplementary data 2 [file mmc2.pdf]

Fig S3

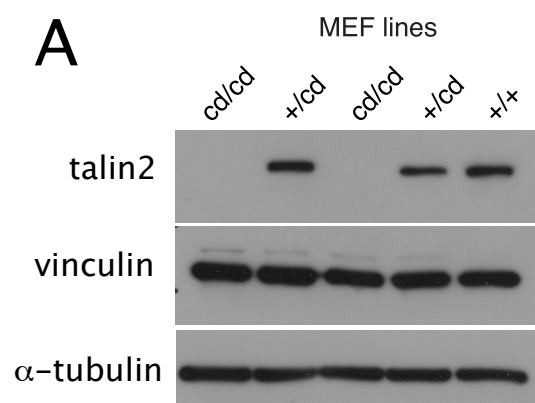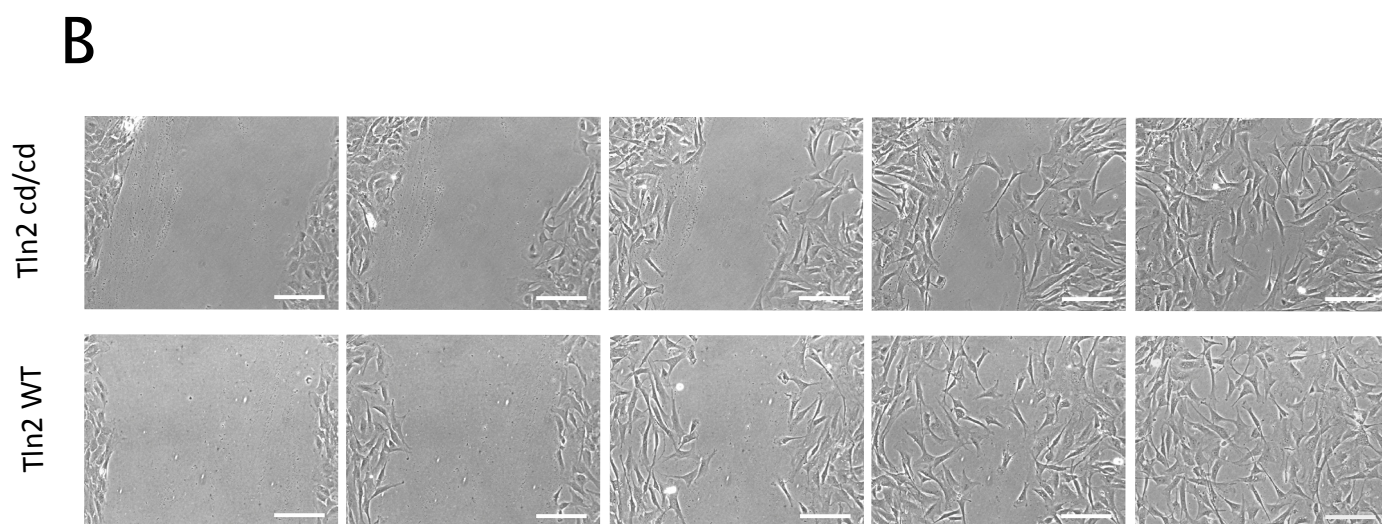

Supplement: Supplementary data 3 [file mmc3.pdf]

Fig S4

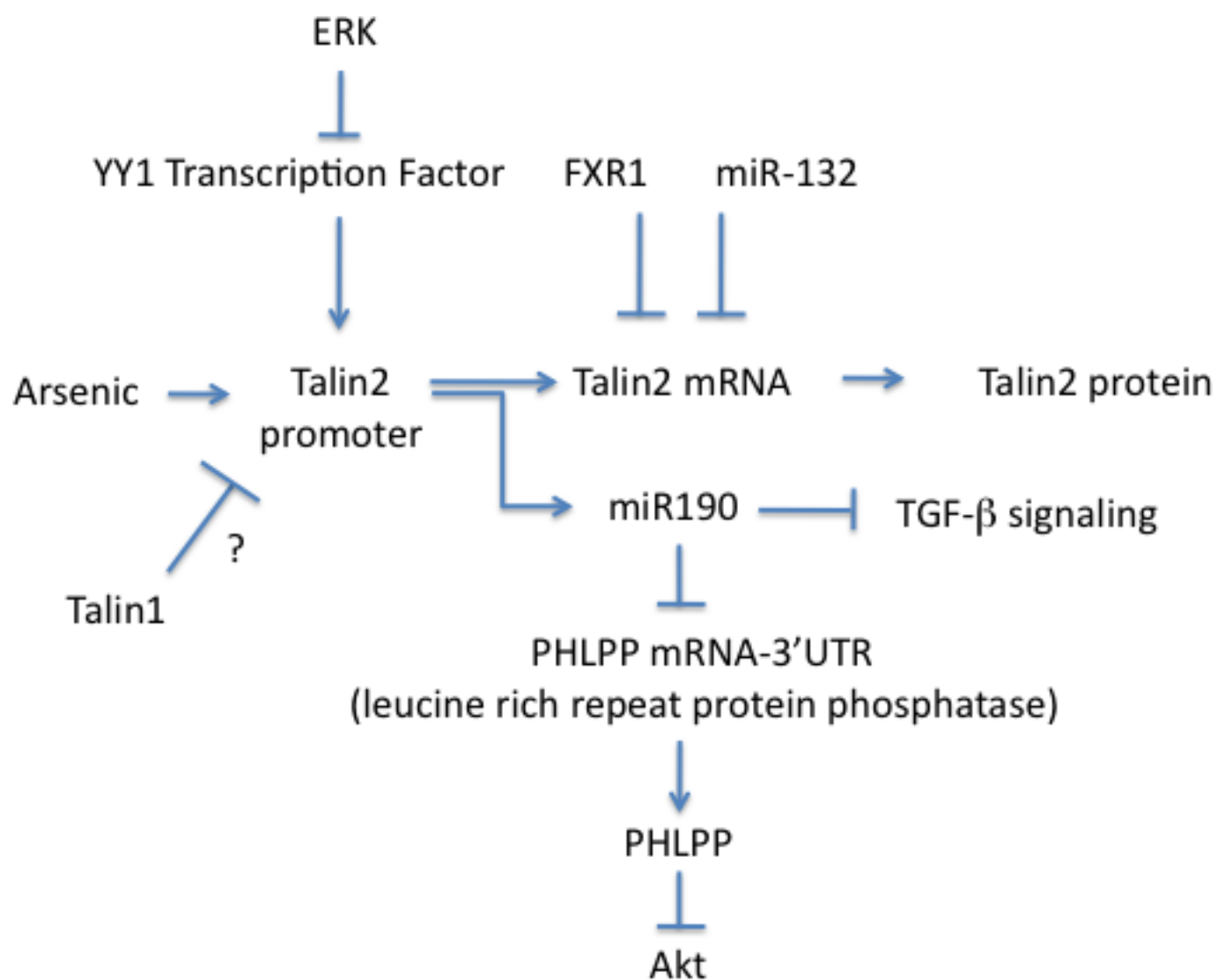

Supplement: Supplementary data 4 [file mmc4.pdf]
