# Supplementary material for: Mice carrying a complete deletion of the talin2 coding sequence are viable and fertile
Source: Biochem Biophys Res Commun. 2012 Sep 21;426(2-3):190–5. doi: 10.1016/j.bbrc.2012.08.061 (PMC3485561; doi:10.1016/j.bbrc.2012.08.061)
Supplement: Supplementary data 5 [file mmc5.docx]

**SUPPLEMENTARY MATERIAL AND METHODS**

#### Deletion of the Tln2 gene in ES cells

#### Recombinant mouse ES clone (#261) carrying a floxed Tln2 exon 1 (LoxP-Neo-LoxP-Exon1-LoxP) [1] was used to target the 3’ end of the Tln2 gene and to delete the region between the first (Exon 1) and last (Exon 56) coding exons. ES #261 cells were grown under standard conditions [2; 3] in the presence of 250ug/ml Neomycin on mitomycin-inactivated Neomycin-resistant feeder fibroblasts produced in-house. The 3’ targeting vector pTKO7 contained the following elements; mouse tln2 left arm, thymidine kinase driven by the PGK promoter, a hygromycin resistance gene driven by the EM7 and PGK promoters, a loxP site and a mouse tln2 right arm. The construct was linearised with NotI, and electroporated (30 µg) into ES #261 cells at 240V and 500μF as previously described [3]. Cells were plated onto mitomycin-inactivated hygromycin resistant fibroblasts and selected for vector integration in the presence of hygromycin B (150 µg/ml) for 8 days. Resistant colonies were picked and expanded in 96-well plates and screened by long range PCR (Bio-X-Long, Bioline), according to the manufacturer’s instructions, using one primer in the pTKO7 targeting vector and one in the Tln2 gene outside of the arms present in the targeting vector. Left arm homologous recombination: primers 5’LAForward (5’-GTACCATGCAGTTTCTCTTTGCG-3’) and 5’HygReverse (5’-GTTCTAATTCCATCAGAAGCTGATC-3’). Right arm homologous recombination: primers 3’HygForward (5’-TAAACTCCTCTTCAGACCATAACTTCG-3’) and 3’RAReverse (5’-AGGGTTTGTCTTCCCTGAATTGTGAC-3’).

Southern blot hybridisation of *Sac*I-digested genomic DNA was used to confirm correct recombination, using probes in the vicinity of the homologous arms and the HSV-TK cassette (Fig. S1A). Probes were generated by PCR amplification of 129/sv mouse genomic DNA or pTKO7 DNA (probe C): Probe A (5’ of Left Homology Arm): primers 5’ProbeForward (5’-TCCACTTACTCCTTGCCCTG-3’ and 5’ProbeReverse (5’-CCCACCTAACCCTGACCACT-3’). Probe B (3’ of right Homology Arm): primers 3’ProbeForward (5’-AGATGGTCACAATTCAGGGAAGA-3’) and 3’ProbeReverse (5’-TGTACTTTATATCTACCTGTGTGTCTTG-3’). Probe C (HSV-TK): primers TKForward (5’-ATACCGCACCGTATTGGCAAGCA-3’) and TKReverse (5’-AAACCACCACCACGCAACTGCTG-3’).

#### Three clones that were correctly targeted (#369, 374 and 397) were expanded and electroporated with 20 µg of the Cre recombinase-expressing plasmid pCre-Pac. Transient puromycin selection (2μg/ml) was applied immediately after transfection for 36 hours to enrich for transfected cells while avoiding integration of the Cre vector. Cells from each of the original clones were kept separate in order to obtain independent deletion events, and one clone per dish was subsequently selected for analysis. Cre-mediated genomic deletion of the complete Tln2 coding region will only occur in cells where the 3’-targeting vector has integrated in cis with the floxed Exon1 allele, and will result in a loss of the 3’-HSV-tk cassette (Fig. S1A). Therefore, ES cells that have undergone the required deletion were identified by selection for Ganciclovir (2μM) resistance. Resistant colonies were screened both by PCR using primers 3 and 4 outside the deleted region (Fig. S1A) (5’-TGTACTTTATATCTACCTGTGTGTCTTG-3’ and 5’-ATAAAGCCATCTGCAACACAGCAA-3’) and by Southern blot hybridization with probe A, B and C in order to identify those carrying the designed genomic deletion (Fig S1A,B).

#### Generation of mice carrying the Tln2 genomic deletion

All procedures on mice were performed in accordance with the UK Animals (Scientific Procedures) Act 1986. Correctly targeted mouse ES cells (369.3, 369.5, 374.1, 374.2, 374.4 and 397.3; Fig S1B) were injected into C57BL/6 blastocysts, and germline transmission from male chimeras assessed through the presence of Agouti pups in the offspring. These mice were genotyped for complete Tln2 deletion (Tln2^cd^), and mice heterozygous for this allele were intercrossed to generate the homozygous deletion mice (Tln2^cd/cd^). All the Tln2^cd^ mice described herein have undergone 2 generations of out-breeding onto the C57Bl/6 genetic background and therefore have a mixed 129/SvJ and C57Bl/6 genetic makeup. The official nomenclature for this allele is Tln2^tm2Crit^.

#### Mouse genotyping

Genomic DNA was extracted from ear notches as previously described [4]. Genotyping was performed in two separate reactions using a primer pair detecting the deletion (primers 1 and 2) (Fig. S1A) and a primer pair detecting Tln2 exon 1. This assay discriminates between Tln2^+/cd^ (deletion and exon 1 positive), Tln2^cd/cd^ (deletion positive and exon 1 negative) and wild-type (deletion negative and exon 1 positive) genotypes.

**Western blotting of embryo and tissue lysates**

Embryos and tissues dissected from genotyped animals were homogenized with a Polytron tissue homogenize in the presence of a cocktail of protease inhibitors (Calbiochem, San Diego, CA, USA; Set III; 1:100 dilution) plus a calpain II inhibitor (E64d; Sigma, St Louis, MO, USA; 1 μg·mL^−1^), and aliquots (40 μg protein) denatured at 100 °C for 2 min in the presence of 5 mm Tris/HCl (pH 6.8), 4% glycerol, 1.6% SDS, 2% bromophenol blue, and 1%β-mercaptoethanol. Proteins were resolved by SDS/PAGE (6% gels) and electroblotted onto Hybond P membranes using wet transfer in 2.5 mM Tris base and 9.6 mM glycine. Excess protein binding sites were blocked with 5% dried milk in NaCl/Tris (pH 7.4). Talin1 and talin2 were detected using isoform-specific monoclonals as described in Methods.

#### Phenotypic analysis of Tln2^cd/cd^ cells

#### Mouse embryo fibroblasts (MEFs) were prepared using standard procedures [5]. For immunofluorescence studies, cells were cultured for 24 h on glass coverslips, fixed and permeabilized in one step with ice-cold methanol for 1 min. Cells were then incubated with 2.5% normal goat serum and 2.5% normal mouse serum for 15 min before staining in 1% BSA in PBS-ME. Talin1 and talin2 were visualised with monoclonal antibodies 97H6 and 68E7, respectively [6] and F-actin with an anti-actin antibody (Sigma, 1:150). Alexa-488 or Alexa-594 coupled secondary antibodies (Molecular Probes) were used at a dilution of 1:200. Epifluorescence images were taken with a 40x oil immersion objective on an inverted Nikon TE300 microscope equipped with a Hamamatsu ORCA-ER digital camera and an X-cite 120 fluorescence illumination system controlled by Improvision's Openlab software. For time-lapse experiments, the temperature was kept at 37 °C in an atmosphere containing ~5% CO_2_. For confocal laser-scanning microscopy, either a Leica TCS SP5 system consisting of a Leica DMI-6000 CS inverted microscope.

#### Muscle immuno-histochemistry

Immuno-histochemistry on muscle was carried out as described [7; 8] using the following antibodies: monoclonal against vinculin (Sigma), MHCf (Sigma), rabbit polyclonal against α7 integrin (kindly provided by U. Meyer, University of East Anglia, Norwich, UK) and laminin α2 (Chemicon). Polyclonal antibodies specific for talin 1 and 2 have been described previously [7], and the epitopes correspond to residues 1830-1850 (talin 1) and 940-957 (talin 2). To determine the number of muscle fibers with central nuclei, random areas across the gastrocnemius were photographed and nuclei quantified. Three mice per genotype were analyzed, 9 fields per sample. The mean (±s.d.) was determined, and significance of the results assessed using a Student's t-test.

**REFERENCES**

[1] F.J. Conti, S.J. Monkley, M.R. Wood, D.R. Critchley, and U. Muller, Talin 1 and 2 are required for myoblast fusion, sarcomere assembly and the maintenance of myotendinous junctions. Development 136 (2009) 3597-606.

[2] E. Debrand, Y. El Jai, L. Spence, N. Bate, U. Praekelt, C.A. Pritchard, S.J. Monkley, and D.R. Critchley, Talin 2 is a large and complex gene encoding multiple transcripts and protein isoforms. FEBS J 276 (2009) 1610-28.

[3] S.J. Monkley, X.-H. Zho, S.J. Kinston, S.M. Giblett, L. Hemmings, H. Priddle, J.E. Brown, C.A. Pritchard, D.R. Critchley, and R. Fassler, Disruption of the talin gene arrests mouse development at the gastrulation stage. Dev. Dynamics 219 (2000) 560-574.

[4] G.E. Truett, P. Heeger, R.L. Mynatt, A.A. Truett, J.A. Walker, and M.L. Warman, Preparation of PCR-quality mouse genomic DNA with hot sodium hydroxide and tris (HotSHOT). BioTechniques 29 (2000) 52, 54.

[5] K. Mercer, S. Giblett, S. Green, D. Lloyd, S. DaRocha Dias, M. Plumb, R. Marais, and C. Pritchard, Expression of endogenous oncogenic V600EB-raf induces proliferation and developmental defects in mice and transformation of primary fibroblasts. Cancer Res 65 (2005) 11493-500.

[6] U. Praekelt, P.M. Kopp, K. Rehm, S. Linder, N. Bate, B. Patel, E. Debrand, A.M. Manso, R.S. Ross, F. Conti, M.Z. Zhang, R.C. Harris, R. Zent, D.R. Critchley, and S.J. Monkley, New isoform-specific monoclonal antibodies reveal different sub-cellular localisations for talin1 and talin2. Eur J Cell Biol 91 (2012) 180-91.

[7] F.J. Conti, A. Felder, S. Monkley, M. Schwander, M.R. Wood, R. Lieber, D. Critchley, and U. Muller, Progressive myopathy and defects in the maintenance of myotendinous junctions in mice that lack talin 1 in skeletal muscle. Development 135 (2008) 2043-53.

[8] M. Schwander, M. Leu, M. Stumm, O.M. Dorchies, U.T. Ruegg, J. Schittny, and U. Muller, Beta1 integrins regulate myoblast fusion and sarcomere assembly. Developmental cell 4 (2003) 673-85.

**SUPPLEMENTARY FIGURE LEGENDS**

**Fig. S1.** **Deletion of the complete Tln2 coding sequence**. (**A**) The 5’ Tln2 targeted allele possesses loxP sites (red arrowheads) flanking the first coding exon (exon 1) plus a floxed Neo gene. ES cells possessing this allele were transfected with a 3’ targeting vector that introduces hygromycin (HYG) and thymidine kinase (TK) genes followed by a loxP site just downstream of the last coding exon (exon 56). Deletion of the entire Tln2 coding sequence was achieved by transfecting the double-targeted ES cells with Cre recombinase to produce the deleted allele (cis). A second possible outcome of the double targeting and Cre recombination, the duplicated allele, could arise if the 2 targeting events occured on different chromosomes (trans). The position of the probes used for Southern blotting are shown above each allele A (blue square), B (green square) and C (red square). Position of the PCR probes (arrows) are shown below each allele. Cen., centromere; Tel., telomere; **(B)** Southern blots of the various ES cells used in generating the deleted allele. Sac1 genomic digests were blotted and hybridised with either probe A (top panel) or probe B (bottom panel). ES cell lines 261 (the parental ES clone possessing the 5’ targeted allele) was transfected with the 3’ targeting vector, and selected clones subsequently screened by Southern blotting. Clones 369, 374 and 397 contain the 3’ targeted allele as demonstrated by the presence of a 11.2kb band with probe A and 8.9kb band with probe B. These ES lines were subsequently transfected with a Cre recombinase expression construct to generate sub-lines 369.3, 369.5, 374.1, 374.2, 374.4 and 397.3. These all possess the deleted allele as shown by loss of the 11.2kb band (probe A) and 8.9kb band (probe B) and appearance of the 6kb band with probe B.

**Fig. S2. Weight of organs in Tln2^cd/cd^ and wild-type mice are similar**

Tln2^+/cd^ animals were intercrossed and organs dissected from adult male mice with the genotypes indicated. L - left, R – right organ.

**Fig. S3. Talin2 null and wild-type MEFs show similar rates of wound closure.**

**(A)** MEFs with the genotypes indicated were Western blotted for talin2. Tln2^cd/cd^ MEFs contain no talin2 but express normal levels of vinculin. α-Tubulin was used as a loading control. (B) MEFs with the genotypes indicated were grown to 80% confluence and the monolayers wounded with a pipette tip. Wound closure was followed by phase contrast time lapse microscopy.

**Fig. S4. Pathways regulating talin2 expression**
